# Reply to: The Epimed Monitor ICU Database^®^: a cloud-based national registry for adult intensive care unit patients in Brazil

**DOI:** 10.5935/0103-507X.20180048

**Published:** 2018

**Authors:** Fernando Godinho Zampieri, Márcio Soares

**Affiliations:** Instituto de Pesquisa, HCor-Hospital do Coração - São Paulo (SP), Brasil.; Instituto D'Or de Pesquisa e Ensino - Rio de Janeiro (RJ), Brasil.; Epimed Solutions - Rio de Janeiro (RJ), Brasil.

We greatly appreciate the comments from Haniffa et al.^(1)^ as well as we are grateful for sharing with the *Revista Brasileira de Terapia Intensiva* readers their experiences with the Sri-Lanka National Registry. They also describe several strengths and challenges in implementing national-level quality improvement initiatives in low-middle income countries. We concur with the authors that this represents a crucial and important step to improve care and efficiency in resource allocation and to foster international collaboration in research among these countries.

*Fernando Godinho Zampieri* *Research Institute, HCor-Hospital do Coração - São
Paulo (SP), Brazil.* *Márcio Soares* *Instituto D'Or de Pesquisa e Ensino - Rio de Janeiro (RJ), Brazil.* *Epimed Solutions - Rio de Janeiro (RJ), Brazil.*
